# Circulating Liver-enriched Antimicrobial Peptide-2 Decreases During Male Puberty

**DOI:** 10.1210/jendso/bvac013

**Published:** 2022-02-08

**Authors:** Tero Varimo, Päivi J Miettinen, Kirsi Vaaralahti, Jorma Toppari, Hanna Huopio, Raimo Voutilainen, Sirpa Tenhola, Matti Hero, Taneli Raivio

**Affiliations:** 1 New Children’s Hospital, Helsinki University Hospital, Pediatric Research Center, FI-00029 HUS, Finland; 2 Stem Cells and Metabolism Research Program, Research Programs Unit, Faculty of Medicine, University of Helsinki, Helsinki FI-00014, Finland; 3 Department of Physiology, Faculty of Medicine, University of Helsinki, FI-00014 Helsinki, Finland; 4 Institute of Biomedicine, Research Centre for Integrative Physiology and Pharmacology, and Centre for Population Health Research, University of Turku, FI-20014 Turku, Finland; 5 Kuopio University Hospital, University of Eastern Finland, FI-70210 Kuopio, Finland; 6 Kymenlaakso Central Hospital, FI-48210 Kotka, Finland

**Keywords:** liver-enriched antimicrobial peptide-2, acylated ghrelin, puberty, testosterone

## Abstract

**Context:**

Circulating levels of liver-enriched antimicrobial peptide 2 (LEAP2), a ghrelin receptor antagonist, decrease under caloric restriction and increase in obesity. The role of LEAP2 in male puberty, a phase with accelerated energy demand, is unclear.

**Objective:**

This work aimed to investigate whether circulating LEAP2 levels are downregulated in boys following the onset of puberty to respond to the energy need required for growth.

**Methods:**

We determined circulating LEAP2 levels in 28 boys with constitutional delay of growth and puberty (CDGP) who participated in a randomized controlled trial (NCT01797718), and were treated with letrozole (n = 15) or intramuscular low-dose testosterone (T) (n = 13) for 6 months. Blood sampling and dual-energy x-ray absorptiometry–measured body composition were performed at 0-, 6-, and 12-month visits.

**Results:**

Serum LEAP2 levels decreased statistically significantly during pubertal progression (0-6 months: mean decrease –4.3 [10.3] ng/mL, *P* = .036 and 0-12 months: –3.9 [9.3] ng/mL, *P* = .033). Between 0 and 6 months, the changes in serum LEAP2 levels correlated positively with changes in percentage of body fat (*r*_s_ = 0.48, *P* = .011), and negatively with growth velocity and estradiol levels (*r*_s_ = –0.43, *P* = .022, *r*_s_ = –0.55, *P* = .003, respectively). In the T group only, the changes in serum LEAP2 correlated negatively with changes in T and estradiol levels. Between 0 and 12 months, the change in LEAP2 levels correlated negatively with the change in high-density lipoprotein levels (*r*_s_ = –0.44, *P* = .022) and positively with the change in insulin (*r*_s_ = 0.50, *P* = .009) and HOMA-IR (r_s_ = 0.51, *P* = .007) levels.

**Conclusion:**

Circulating LEAP2 levels decreased after induction of puberty reciprocally with increased growth rate and energy demand, reflecting the metabolic state of the adolescent. Further, the results suggest that estradiol levels may have a permissive role in downregulating circulating LEAP2 levels.

Puberty is associated with increased energy consumption due to both accelerated height velocity and gain in muscle and bone mass [1]. These anabolic changes are more profound in males than in females and are especially attributed to testosterone (T), which increases, directly or indirectly, growth velocity, bone mineral acquisition, and lean mass, and reduces fat mass [2, 3]. To meet these anabolic changes during puberty, energy intake needs to increase accordingly. Ghrelin is a key driver of energy seeking and storage that reverses energy deficit during times of metabolic need [4, 5]. The acylated form of ghrelin (acyl-ghrelin) binds to the growth hormone secretagogue receptor (GHSR) in the hypothalamic arcuate nucleus, where it regulates neuropeptide Y production to increase food intake, weight gain, and the secretion of GH [6-9]. Regarding GH secretion and height growth, the constitutional activity of GHSR may be more important than ghrelin stimulated, as highlighted by findings in short individuals carrying an Ala204Glu mutation in *GHSR* [10]. The connection between somatic changes during puberty and GHSR activity has been little studied; however, limited evidence supports the view that variation in *GHSR* may be related to constitutional delay of growth and puberty (CDGP), a common form of transient short stature and lean phenotype [11].

Recently, liver-enriched antimicrobial peptide-2 (LEAP2), produced in the liver and small intestine, was shown to antagonize GHSR (ie, ghrelin receptor) and thereby prevent the actions of ghrelin on GH secretion and increased food intake [12]. Accordingly, circulating LEAP2 and acyl-ghrelin correlated negatively, and serum LEAP2 levels are elevated in obesity and decreased in energy-deficient states, which then allows the action of the acyl-ghrelin [13]. Thus, there exist 2 circulating ligands for the GHSR with opposing effects that tightly regulate the activity of GHSR (reviewed in [14]). Of note, LEAP2 downregulates both constitutional and ghrelin-evoked activity of GHSR [15]. Currently, only one cross-sectional study has examined circulating LEAP2 levels in pubertal individuals; the levels were higher in pubertal girls than in prepubertal participants; however, similar results were not observed in boys [16]. Taking into account the interindividual variation in LEAP2 levels and the cross-sectional design in that study, it is obvious that the role of LEAP2 in the regulation of somatic changes of puberty is unclear and longitudinal analyses on this topic are needed.

We hypothesized that circulating LEAP2 levels are downregulated in boys following the onset of puberty to respond to the energy need required for growth. To test this hypothesis we investigated longitudinal changes in serum LEAP2 levels in 28 boys with CDGP, who were treated with either low-dose T or aromatase inhibitor letrozole for 6 months to promote the progression of puberty and followed up to 12 months [17].

## Materials and Methods

This study included 28 boys with CDGP who participated in a randomized controlled trial in 4 Finnish pediatric endocrinology outpatient clinics between 2013 and 2017 [17]. In the trial, the boys were randomly assigned to receive either aromatase inhibitor letrozole (2.5 mg/d) (n = 15) or intramuscular T (1 mg/kg/every 4 weeks) (n = 13) for 6 months, and the study protocol included study visits at 0, 6, and 12 months. At each visit, height, weight, growth velocity, and testicular volume were measured, and pubertal stage was recorded. These values have been reported previously [17]. We calculated the age-adjusted body mass index (BMI) values (ISO-BMI) from growth measurements by using Finnish reference data [18]. At the start of the study, the mean (SD) ISO-BMI was 21.9 (4.9).

Fasting morning serum samples were taken at 0-, 6-, and 12-month visits, and serum samples were stored at –80 °C. Testosterone, estradiol, luteinizing hormone, follicle-stimulating hormone, insulin-like growth factor-1 (IGF-1), and inhibin B levels were determined with routine laboratory techniques (liquid chromatography/mass spectrometric, immunoelectrochemiluminometric, and enzyme-linked immunosorbent assays [ELISAs]), as described previously [17]. Plasma total cholesterol, high-density lipoprotein cholesterol (HDL), low-density lipoprotein cholesterol (LDL), and triglycerides (TGs), insulin, and glucose levels were determined with accredited standard methods at Helsinki University Hospital and Kuopio University Hospital laboratories. The Homeostatic Model Assessment for Insulin Resistance (HOMA-IR) was calculated by using the formula (glucose [mmol/L] × insulin) [mU/L]/22.5 [19]. Hormonal values and markers of lipid and glucose metabolism at each time point are presented in Table 1.

**Table 1.** Hormones and markers of lipid and glucose metabolism at 0-, 6-, and 12-month visits in 28 boys with constitutional delayed of growth and puberty treated with letrozole or testosterone for 6 months

	Letrozole (n = 15)			Testosterone (n = 13)		
	0 mo	6 mo	12 mo	0 mo	6 mo	12 mo
Testicular volume, mL	2.9 (1.2-4.8)	10.1 (3.3-17.8)	12.7 (4.9-18.3)	3.5 (2.2-5.0)	5.7 (3.8-8.3)	9.7 (5.4-12.9)
Testosterone, nmol/L	1.9 (0.7-4.5)	30.2 (5.7-59.1)	10.2 (4.7-18.3)	2.2 (0.3-4.5)	5.7 (2.2-10.8)	11.5 (6.5-17.2)
Estradiol, pmol/L	9.7 (5.0-19.0)	15.7 (5.0-95.0)	32.6 (11.0-52.0)	14.0 (5.0-39.0)	22.0 (6.8-74.0)	39.6 (14.0-74.0)
Inhibin B, ng/L	174 (101-316)	211 (141-352)	223 (135-383)	201 (87-407)	167 (60-425)	211 (57-465)
IGF-1, nmol/L	31 (18-55)	32 (19-43)	45 (24-73)	36 (21-63)	50 (27-79)	49 (27-76)
Total cholesterol, mmol/L	4.4 (3.5-6.3)	3.9 (3.2-4.9)	4.1 (3.2-6.3)	4.2 (3.2-5.2)	3.9 (3.2-4.8)	3.8 (3.0-4.2)
HDL cholesterol, mmol/L	1.7 (0.8-2.4)	1.3 (0.8-2.2)	1.6 (0.8-2.5)	1.6 (1.1-2.8)	1.4 (1.0-2.7)	1.5 (1.0-2.1)
LDL cholesterol, mmol/L	2.7 (1.6-4.2)	2.5 (1.8-3.6)	2.4 (1.7-4.4)	2.5 (1.2-3.9)	2.3 (1.4-3.4)	2.1 (1.4-2.8)
Triglycerides, mmol/L	0.8 (0.3-3.1)	0.9 (0.4-2.4)	0.8 (0.4-2.4)	0.9 (0.4-1.7)	1.0 (0.4-1.9)	1.0 (0.3-1.9)
Glucose, mmol/L	5.6 (5.3-5.8)	5.5 (5.3-5.7)	5.6 (5.3-5.9)	5.3 (4.8-5.9)	5.4 (4.6-5.9)	5.3 (4.9-5.9)
Insulin, mU/L	11.6 (3.8-41.4)	10.4 (3.2-26.7)	16.6 (4.3-45.6)	11.7 (5.0-22.0)	17.1 (3.9-34.8)	14.8 (0.3-64.6)
HOMA-IR	3.0 (0.8-11.6)	2.6 (0.8-6.9)	4.4 (0.9-14.0)	2.8 (1.1-5.8)	4.2 (0.8-9.0)	3.5 (0.1-15.8)
Growth velocity, cm/y	3.9 (1.7-5.3)	6.3 (3.6-11.0)	7.0 (4.1-11.6)	4.5 (2.5-8.7)	8.2 (5.2-13.0)	7.9 (5.0-10.5)

Mean (range).

Abbreviations: HDL, high-density lipoprotein; HOMA-IR, Homeostatic Model Assessment for Insulin Resistance; IGF-1, insulin-like growth factor-1; LDL, low-density lipoprotein.

LEAP2 levels were assessed using a specific enzyme immunoassay for human and mouse LEAP2 detection (EK-075-40, Phoenix Pharmaceuticals) according to the manufacturer’s instructions. This LEAP2 ELISA assay kit has recently been validated in human samples [13]. For LEAP2 estimation, serum samples were diluted 10 times in the assay buffer. Intra-assay and interassay coefficients of variation (CVs) listed by the manufacturer were less than 10% and less than 15%, respectively. In our hands, intra-assay CV was less than 6% at 10 ng/mL, and interassay CV was 17% at 10 ng/mL.

Serum acylated ghrelin was analyzed using the human acylated ghrelin ELISA commercial kit (Human Acylated Ghrelin Easy Sampling Enzyme Immunoassay Kit, Bertin Pharma) immediately after thawing the samples for the first time. All samples were diluted (in 1:2) to bring the analyte level to the measuring range of the assay, as instructed by the manufacturer. The final results were calculated by multiplying the results by the dilution factor. As reported by the manufacturer, the limit of detection in the samples is 4 pg/mL. Longitudinal samples from each individual were assayed on the same assay plate as singleton measurements (within-assay CV was 7.1% and between-assay CV was 4.2%).

At the 0-, 6-, and 12-month visits, fat mass, percentage body fat, and lean mass (ie, body composition) were measured with dual-energy x-ray absorptiometry (Lunar Prodigy; GE Healthcare, or Hologic Discovery A; Hologic). Both treatments decreased fat mass and percentage body fat, and induced an increase in lean mass, as previously reported [17]. Thus, we pooled the 2 treatment groups when investigating the relationship between circulating LEAP2 or acyl-ghrelin and markers of body composition.

The study was registered with ClinicalTrials.gov (NCT01797718), and the Finnish National Committee on Medical Research Ethics and the Finnish Medicines Agency approved the study. A written consent was obtained from all participants.

### Statistical Analyses

The data are presented with mean and SD unless otherwise noted. Analyses were performed with SPSS for Windows (version 22.2). Between-group comparisons were performed with an independent-samples *t* test, and within-group comparisons with a simple *t* test. Correlations between LEAP2 or acyl-ghrelin levels and markers of glucose and lipid metabolism, body composition, and hormonal markers of puberty were evaluated with Spearman rank correlation. In the analyses, delta (Δ) signifies the change: value at 6 months (or 12 months) minus value at 0 months. This was an exploratory study, and the level of statistical significance was set to a *P* value less than .05.

## Results

### Overall Change in Liver-enriched Antimicrobial Peptide 2 Levels

**Figure 1.**
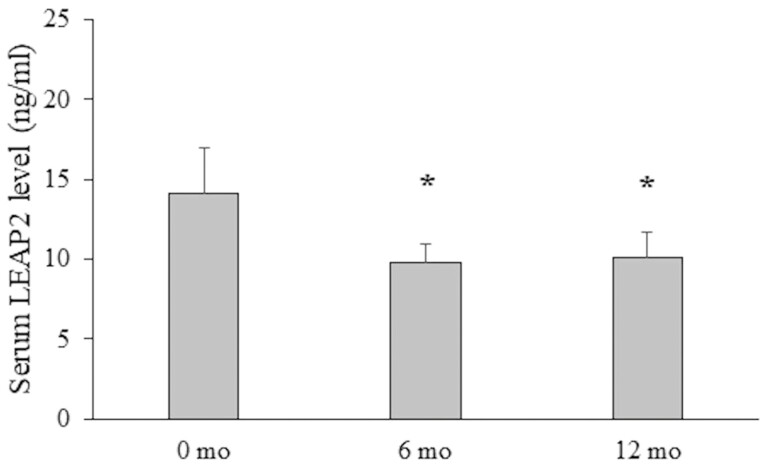
The mean and SEM of serum liver-enriched antimicrobial peptide 2 (LEAP2) levels in 28 boys with constitutional delay of growth and puberty at 0, 6, and 12 month visits of the study. For the initial 6 months, the boys were treated with either letrozole or testosterone. *P less than .05 for the change between 0 and 6 and 0 and 12 months.

Serum LEAP2 levels declined between 0 and 6 months (mean change, –4.3 [10.3] ng/mL, *P* = .036) and between 0 and 12 months (–3.9 [9.3] ng/mL, *P* = .033) (Fig. 1). In both treatment groups, however, the decline in LEAP2 levels did not reach statistical significance (*P* = .17-.30).

### Correlation Between Liver-enriched Antimicrobial Peptide 2 and Markers of Body Composition

**Figure 2.**
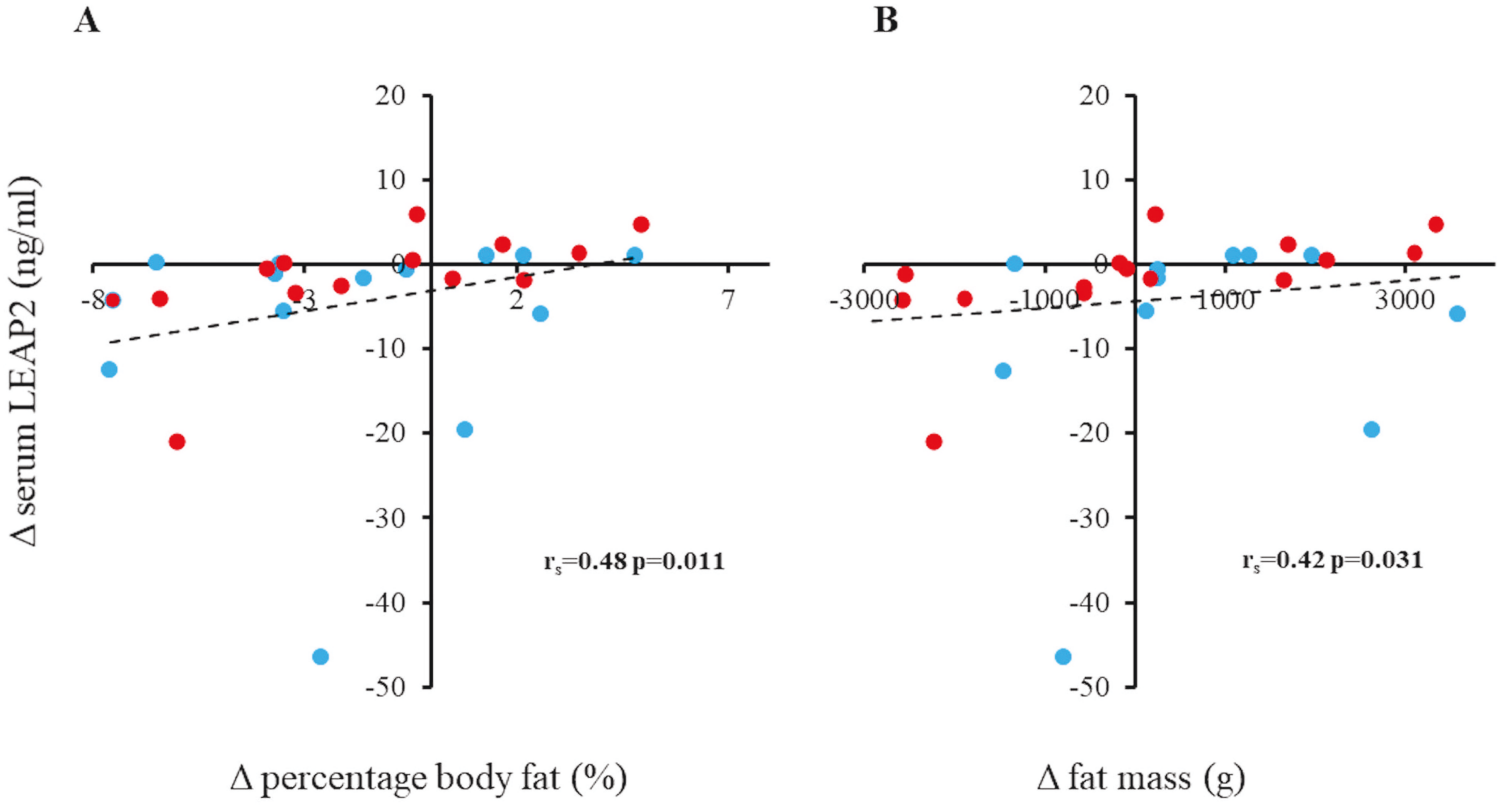
Correlations between Δliver-enriched antimicrobial peptide 2 (LEAP2) levels and A, Δpercentage body fat, and B, Δfat mass in 28 boys with constitutional delay of growth and puberty who were treated with either testosterone (blue) or letrozole (red) for 6 months. Δ, level at 6 months minus level at 0 months.

At the start of the study, LEAP2 levels correlated positively with ISO-BMI values and percentage body fat and fat mass (*r*_s_ = 0.48, *P* = .009; *r*_s_ = 0.61, *P* = .001; and *r*_s_ = 0.59, *P* = .001, respectively). Between 0 and 6 months, ΔLEAP2 correlated positively with Δfat mass and Δpercentage body fat, and negatively with Δgrowth velocity (Fig. 2; Table 2).

**Table 2.** Correlations between treatment-induced changes in LEAP2 and clinical markers and markers of lipid and glucose metabolism between 0 and 6 months and 0 and 12 months of follow-up

0-6 mo	ΔLEAP2
Δ	
Growth velocity, cm/v	***r***_**s**_ ** = –0.43, *P* = .022**
Glucose	*r*_s_ = 0.09, *P* = .63
Insulin	*r*_s_ = 0.35, *P* = .07
HOMA-IR	*r*_s_ = 0.30, *P* = .13
Total cholesterol	*r*_s_ = –0.13, *P* = .53
HDL	***r***_**s**_ ** = –0.55, *P* = .003**
LDL	*r*_s_ = 0.004, *P* = .98
TGs	***r***_**s**_ ** = 0.55, *P* = .003**
**0-12 mo**	**ΔLEAP2**
Δ	
Growth velocity, cm/v	*r*_s_ = 0.02, *P* = .92
Glucose	*r*_s_ = 0.30, *P* = .13
Insulin	***r***_**s**_ ** = 0.50, *P* = .009**
HOMA-IR	***r***_**s**_ ** = 0.51, *P* = .007**
Total cholesterol	*r*_s_ = –0.28, *P* = .15
HDL	***r***_**s**_ ** = –0.44, *P* = .022**
LDL	*r*_s_ = –0.25, *P* = .21
TGs	*r*_s_ = 0.36, *P* = .06

All *P*-values less than 0.05 could be bolded.

Abbreviations: Δ, value at 6 months (or 12 months) minus value at 0 months; HDL, high-density lipoprotein; HOMA-IR, Homeostatic Model Assessment for Insulin Resistance; LDL, low-density lipoprotein; LEAP2, liver-enriched antimicrobial peptide 2; TGs, triglycerides.

### Correlations Between Liver-enriched Antimicrobial Peptide 2 Levels and Markers of Lipid and Glucose Metabolism

At the start of study, LEAP2 levels correlated positively with TGs (*r*_s_ = 0.48, *P* = .012) and insulin levels (*r*_s_ = 0.48, *P* = .012) and HOMA-IR (*r*_s_ = 0.44, *P* = .023) and negatively with HDL levels (*r*_s_ = –0.63, *P* < .001). ΔLEAP2 correlated positively with ΔTG levels and negatively with ΔHDL levels (see Table 2). Between 0 and 12 months, ΔLEAP2 correlated negatively with ΔHDL levels and positively with Δinsulin and ΔHOMA-IR levels (see Table 2).

### Correlations Between Liver-enriched Antimicrobial Peptide 2 Levels and Hormonal Markers of Puberty

**Figure 3.**
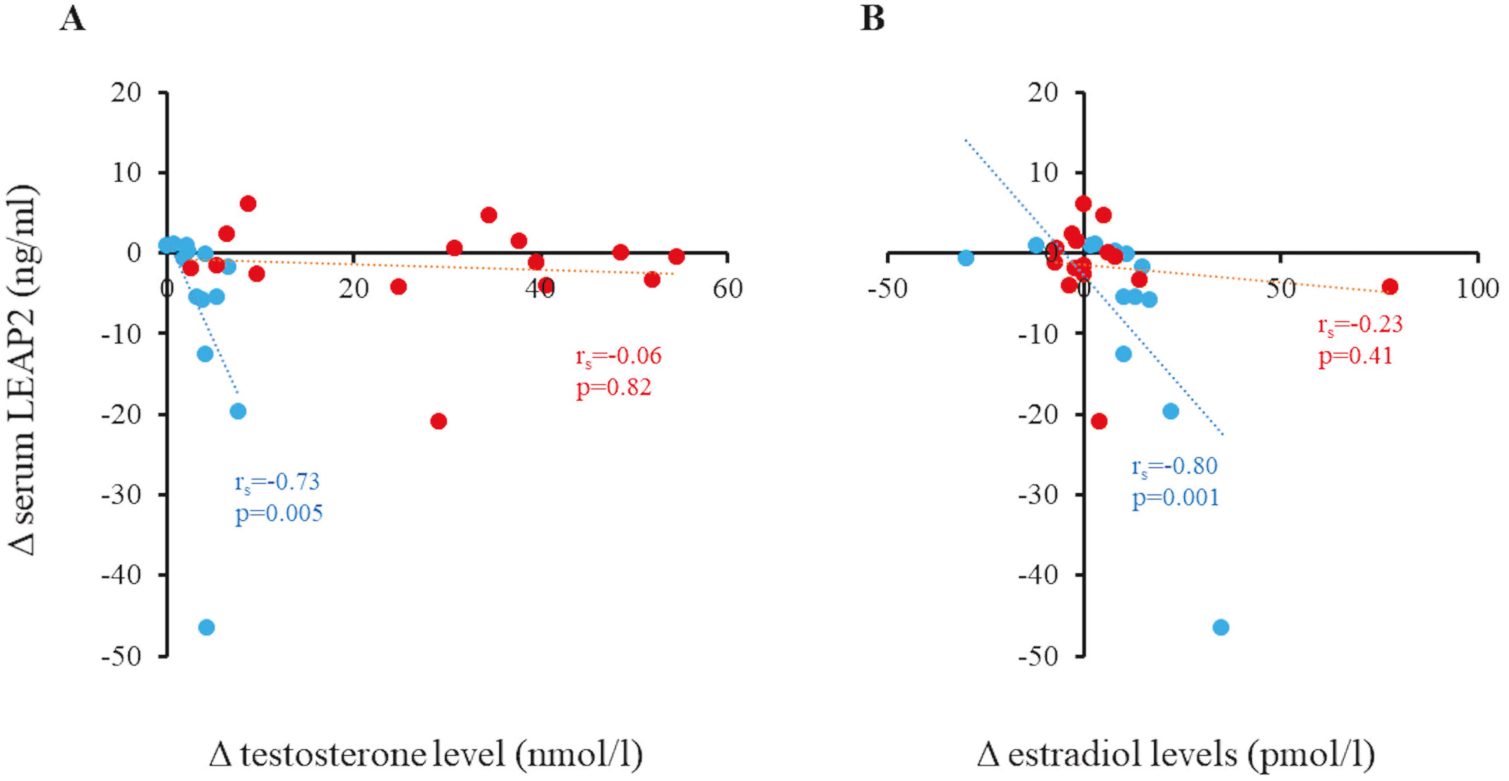
Correlations between Δliver-enriched antimicrobial peptide 2 (LEAP2) levels and A, Δtestosterone, and B, Δestradiol levels in 28 boys with constitutional delay of growth and puberty who were treated with either testosterone (blue) or letrozole (red) for 6 months. Δ, level at 6 months minus level at 0 months.

Between 0 and 6 months, ΔLEAP2 in all CDGP boys correlated negatively with the corresponding Δestradiol level (*r*_s_ = –0.55, *P* = .003). However, ΔLEAP2 did not correlate with IGF-1 levels during treatment or follow-up (0-6 months: *r*_s_ = –0.15, *P* = .47; 0-12 months: *r*_s_ = 0.01, *P* = .99). In T-treated boys, ΔLEAP2 correlated negatively with ΔT and Δestradiol (Fig. 3), but not with ΔIGF-1 levels. Noteworthy, similar correlations were not found in letrozole-treated individuals (see Fig. 3).

### Acyl-Ghrelin Levels During Study Period

Overall, in the whole group, acyl-ghrelin levels did not change statistically significantly during the study period (0-6 months: –5.5 pg/mL [22.7], *P* = .26 and 0-12 months: –5.9 pg/mL [40.0], *P* = .47). The mean acyl-ghrelin levels at each time were as follows; 0 months: 54.1 pg/mL (65.2), 6 months: 51.2 pg/mL (56.8), and 12 months: 49.2 pg/mL (53.6). In the T group, Δacyl-ghrelin between 0 and 6 months correlated negatively with ΔHOMA-IR (*r*_s_ = –0.73, *P* = .011) and Δinsulin (*r*_s_ = –0.73, *P* = .011). No statistically significant correlations were found between acyl-ghrelin levels and markers of lipid and glucose metabolism, body composition, and hormonal markers of puberty (*P* = .06-.95). The Acyl-ghrelin/LEAP2 ratio did not change statistically significantly during the study period or correlate statistically significantly with body composition, markers of lipid and glucose metabolism, or hormonal changes of puberty.

## Discussion

We hypothesized that circulating LEAP2 levels decrease in males after the onset of puberty to optimize increase in energy intake to meet the demands brought about by increased growth rate and anabolic changes in body composition [20]. We investigated this longitudinally in a cohort of boys who received puberty-promoting treatment [17]. In accordance with our hypothesis, we found that LEAP2 levels decreased substantially as puberty progressed, and the changes in LEAP2 correlated positively with changes in fat mass and percentage body fat, as evaluated by dual-energy x-ray absorptiometry, and negatively with treatment-induced changes in growth rate. These findings suggest that LEAP2 has a considerable role in the regulation of energy balance during male puberty. Our findings in pubertal boys are in line with the recent findings by Mani et al [13], who reported higher circulating LEAP2 in obese adults and positive correlations between LEAP2 levels and fat mass and visceral adipose tissue. Similar findings have been reported from studies with mice with decreased LEAP2 after diet-induced weight loss and increased LEAP2 after diet-induced obesity [12, 21]. Of note, our findings differ from the study by Barja-Fernández and colleagues [16], who reported no difference in circulating LEAP2 between prepubertal and pubertal boys in a cross-sectional setting . This discrepancy is likely explained by differences in study designs and assessments of study end points. Direct comparison of our results to those by Barja-Fernandez et al requires caution, since boys with CDGP have greater metabolic needs as their total energy expenditure is higher than in age- and height-matched peers [22]. In addition, energy expenditure increases substantially during puberty [20], which highlights the value of a longitudinal study design when addressing the putative role of LEAP2 during puberty.

The negative correlation between growth velocity and LEAP2 level may reflect meaningful downregulation of LEAP2 synthesis in response to increased energy demand, or in causally reverse fashion, increase in growth rate induced by decreased LEAP2 antagonism on GHSR and stimulation of GH secretion. Regarding the latter, systemic injection of LEAP2 has been shown to impair ghrelin-induced GH release in mice, supporting that LEAP2 modulates GH secretion through GHSR in rodents [12]. Interestingly, Shankar et al [23] showed that *Leap2* knockout female mice exhibit exaggerated GH secretion, fat accumulation, and growth. In our study, the changes in LEAP2 did not correlate with those of IGF-1, a marker of GH secretion. Additionally, IGF-1 levels did not correlate with growth velocity [24], raising the doubt that IGF-1 did not adequately reflect GH secretory status in male CDGP patients, in whom pubertal activation of the IGF-1 secretion is blunted when a potent estrogen-suppressive treatment is used. Indeed, serum IGF-1 level depends not only on GH secretory status, but also on childhood protein intake and body composition, with higher levels in children with greater fat and fat-free mass [25, 26]. Overall, the role of LEAP2 in the regulation of the GH–IGF-1 axis in humans is currently unclear. Future studies will clarify whether circulating LEAP2 is another important modulator of the GH–IGF-1 axis activity that is secreted in response to changing energy balance, as has previously been reported on serum fibroblast growth factor 21 [27].

The changes in LEAP2 showed a statistically significant negative correlation with changes in T and estradiol, but only in CDGP boys who received aromatizable T. This finding raises the intriguing question whether in pubertal boys, aromatization of androgens to estrogens and possibly following estrogen-mediated stimulation of GH secretion is a prerequisite for pubertal downregulation of LEAP2. To this end, an enteroendocrine connection between GH secretory status and ghrelin secretion was recently reported, with reduced postprandial ghrelin attenuation in patients with isolated GHD [28]. Recently, a cross-sectional pilot study by Vergani et al [29] evaluated the levels of LEAP2 in adults with GH deficiency, and found a trend toward higher LEAP2 levels in these patients than in healthy controls. Some evidence from a study in rodents also points to estrogen-dependent modulation of sensitivity to ghrelin in males and ovariectomized females [30]. Overall, there are very few data available on the association between estrogen and LEAP2 and further studies are required to clarify this.

The results of our study demonstrated a positive correlation between changes in circulating LEAP2 levels and those in insulin levels and HOMA-IR. Interestingly, the changes in LEAP2 also correlated positively with the changes in TG levels and inversely with the changes in HDL. In line with our results, Mani and colleagues [13] demonstrated that LEAP2 levels correlated positively with HOMA-IR, TGs, plasma glucose, and percentage body fat. These findings corroborate a close endocrine relationship between circulating LEAP2 levels and indices of lipid and glucose metabolism, and suggest that high LEAP2 level in an adolescent boy may serve as an indicator of an unfavorable glucose and lipid metabolism profile.

Neither circulating acyl-ghrelin levels nor acyl-ghrelin/LEAP2 ratio changed substantially during follow-up. At first glance, this is surprising as one would expect opposite changes in acyl-ghrelin and LEAP2 in relation to changing energy demand according to findings reported by Mani et al [13]. Our finding may be related to differences between acyl-ghrelin and LEAP2 in activating the GHSR, as the latter not only competes with acyl-ghrelin for binding sites in GHSR but also reduces the constitutive activity of GHSR in the absence of ghrelin (reviewed in [31]). Thus, our findings may point to a more sensitive role for LEAP2 than acyl-ghrelin as a peripheral signal of energy need during rapid growth. This conclusion naturally requires confirmation in another population of pubertal boys. In boys treated with testosterone, acyl-ghrelin levels correlated negatively with HOMA-IR and insulin levels, which are opposite to the LEAP2 correlations and support the view that LEAP2 and acyl-ghrelin have opposite functions in glucose metabolism. The strength of the present study is the longitudinal design and carefully determined study cohort [17]. Additionally, all blood samples were taken in a standardized fashion after 12 hours of fasting, yet we did not collect data on the prior diet of the participants. Limitations are the lack of an untreated control population, which we have discussed before [17], a limited number of participants (a common challenge in pediatric drug trials), and the lack of leptin levels, which would have permitted the calculation of the leptin/ghrelin ratio. Future studies should investigate the longitudinal changes in LEAP2 in relation to changes in growth rate and body composition in other populations, including boys and girls who progress through normally timed puberty, the association between estradiol and LEAP2 in adolescent girls and boys, the association between GH secretion and LEAP2, and possible differences in LEAP2 levels in different pediatric populations of divergent ethnic origin.

In conclusion, this exploratory study showed that circulating LEAP2 levels decrease in boys with CDGP receiving treatment to induce puberty after the onset of puberty in keeping with increased energy demand. The changes in LEAP2 correlate positively with the changes in fat mass and levels of TGs, insulin, and HOMA-IR, whereas similar correlations were not observed with acyl-ghrelin levels. These findings suggest that changes in LEAP2 reflect changes in the metabolic state of the adolescent. In CDGP boys treated with aromatizable T, the change in LEAP2 levels correlated negatively with the change in estradiol, suggesting a possible permissive role for estradiol in the downregulation of LEAP2.

## Data Availability

The data that support the findings of this study are available from the corresponding author on reasonable request. Data will be shared according to the EU General Data Protection Regulation and national and hospital data protection regulations.

## Abbreviations

BMI: body mass index
CDGP: constitutional delay of growth and puberty
CV: coefficient of variation
ELISA: enzyme-linked immunosorbent assay
GH: growth hormone
GHSR: growth hormone secretagogue receptor
HDL: high-density lipoprotein
HOMA-IR: Homeostatic Model Assessment for Insulin Resistance
IGF-1: insulin-like growth factor-1
LDL: low-density lipoprotein
LEAP2: liver-enriched antimicrobial peptide-2
T: testosterone
TGs: triglycerides

## Clinical Trial Information

ClinicalTrials.gov registration number NCT01797718 (registered February 22, 2013).
